# Small heterodimer partner (SHP) deficiency protects myocardia from lipid accumulation in high fat diet-fed mice

**DOI:** 10.1371/journal.pone.0186021

**Published:** 2017-10-10

**Authors:** Jung Hun Ohn, Ji Yeon Hwang, Min Kyong Moon, Hwa Young Ahn, Hwan Hee Kim, Young Do Koo, Kwang-Il Kim, Hyuk Jae Chang, Hye Seung Lee, Hak Chul Jang, Young Joo Park

**Affiliations:** 1 Department of Internal Medicine, Seoul National University Bundang Hospital, Seongnam, Republic of Korea; 2 Preclinical Research Center, Biomedical Research Institute, Seoul National University Bundang Hospital, Seongnam, Republic of Korea; 3 Department of Internal Medicine, Seoul National University College of Medicine, Seoul, Republic of Korea; 4 Department of Internal Medicine, Boramae Medical Center, Seoul, Republic of Korea; 5 Department of Internal Medicine, Chung-Ang University Hospital, College of Medicine, Chung-Ang University, Seoul, Republic of Korea; 6 Clinical Research Institute, Seoul National University Hospital, Seoul, Republic of Korea; 7 Division of Cardiology, Yonsei Cardiovascular Center, Yonsei University College of Medicine, Seoul, Republic of Korea; 8 Department of Pathology, Seoul National University Bundang Hospital, Seongnam, Republic of Korea; Universite du Quebec a Montreal, CANADA

## Abstract

The small heterodimer partner (SHP) regulates fatty acid oxidation and lipogenesis in the liver by regulating peroxisome proliferator-activated receptor (PPAR) γ expression. SHP is also abundantly expressed in the myocardium. We investigated the effect of SHP expression on myocardia assessing not only heart structure and function but also lipid metabolism and related gene expression in a SHP deletion animal model. Transcriptional profiling with a microarray revealed that genes participating in cell growth, cytokine signalling, phospholipid metabolism, and extracellular matrix are up-regulated in the myocardia of SHP knockout (KO) mice compared to those of wild-type (WT) mice (nominal p value < 0.05). Consistent with these gene expression changes, the left ventricular masses of SHP KO mice were significantly higher than WT mice (76.8 ± 20.5 mg vs. 52.8 ± 6.8 mg, P = 0.0093). After 12 weeks of high fat diet (HFD), SHP KO mice gained less weight and exhibited less elevation in serum-free fatty acid and less ectopic lipid accumulation in the myocardium than WT mice. According to microarray analysis, genes regulated by PPARγ1 and PPARα were down-regulated in myocardia of SHP KO mice compared to their expression in WT mice after HFD, suggesting that the reduction in lipid accumulation in the myocardium resulted from a decrease in lipogenesis regulated by PPARγ. We confirmed the reduced expression of PPARγ1 and PPARα target genes such as CD36, medium-chain acyl-CoA dehydrogenase, long-chain acyl-CoA dehydrogenase, and very long-chain acyl-CoA dehydrogenase by SHP KO after HFD.

## Introduction

The small heterodimer partner (SHP) is an atypical orphan nuclear receptor that regulates the expression of genes involved in glucose, lipid, and bile acid metabolism and plays a key role in metabolic homeostasis [1, 2]. Interestingly, SHP-deficient mice exhibit increased fatty acid oxidation (FAO) and decreased lipogenesis, and thus, are protected from diet-induced hepatic steatosis [3–7]. This is associated with the activation of peroxisome proliferator-activated receptor γ (PPARγ) by SHP [8]. In SHP-deficient mice, very low expression of PPARγ2, a potent lipogenic transcription factor, decreases lipogenesis in the liver [7].

SHP is abundantly expressed not only in the liver but also in the myocardium. The heart is one of the most energy-demanding organs in the body. Heart energy is primarily derived from FAO, and long-chain fatty acids (LCFAs) are the preferred substrate for oxidative phosphorylation in cardiac mitochondria [9, 10]. To date, few studies have investigated the metabolic role of SHP in the myocardium. One study found that the hearts of SHP-deficient mice were hypertrophied and that SHP blocked the development of hypertrophy by interfering with GATA6 signalling [11]. However, it was recently reported that SHP overexpression in cardiomyocytes induced lipid accumulation, insulin resistance, and inflammation [12].

In the present study, we comprehensively profiled gene expression changes in the myocardium via SHP knockout (KO) and investigated the effect of SHP deletion on the myocardium, assessing not only heart structure and function but also lipid metabolism. We found that SHP-deficient mice hearts hypertrophied without functional change and exhibited less ectopic lipid accumulation after a high fat diet (HFD), likely resulting from a reduction in lipogenesis regulated by PPARγ.

## Materials and methods

### Animals

The SHP-deficient mice (SHP KO) were kindly donated from Baylor College of Medicine (Houston, TX, USA) with Dr. Moore’s permission. SHP KO mice were further backcrossed to C57BL/6 mice (WT), which were purchased from Orient (Seongnam, Korea), at our laboratory [7]. All mice were fed an ad libitum laboratory chow diet (CD, Purina irradiated laboratory chow 38057, Purina Korea, Seoul, Korea) prior to the experiments. Five- to Six-week-old male WT and SHP KO mice were fed a HFD (Research Diets D12079B, Research Diets, New Brunswick, NJ, USA) for 12 weeks. The HFD included approximately 4.7 kcal/g, with 17% protein, 40% fat, and 43% carbohydrates. Each group included eight to ten mice, and the experiment was repeated at least three times. All mice were housed in conventional plastic cages with free access to food and water at 23 ± 2°C, 60 ± 10% humidity, and a 12-h light/12-h dark photoperiod. At the end of 12 weeks, mice were anaesthetized with an intraperitoneal injection of a mixture of zolazepam/tiletamine (80 mg/kg; Zoletil 50®, Virbac, France) and xylazine (20 mg/kg; Rompun®, Bayer HealthCare, Germany) for tissue sampling after 8 h of fasting. Whole blood samples were collected and the mice were sacrificed by excision of the heart under deep anaesthesia. All procedures involving the use of laboratory animals were in accordance with the Guide for Standard Operation Procedures, and were performed after receiving approval from the Institutional Animal Care and Use Committee (IACUC) of the Clinical Research Institute, Seoul National University Bundang Hospital (approval No. BA1103-079/021-01).

### Left Ventricle (LV) structure and function

Transthoracic echocardiography (M-mode two-dimensional echocardiography) was performed on anaesthetized mice (1.8% isoflurane, inhalation) using a Philips iE33 ultrasound machine with a 15 mHz liner array transducer, at baseline, 6 weeks, and 12 weeks after HFD feeding by an experienced cardiologist who was blinded to all groups. LV chamber dimensions (LV end-diastolic dimension, LVEDD; LV end-systolic dimension, LVESD), LV wall thicknesses (LV posterior wall, LVPWd; interventricular septum, IVS), fractional shortening (FS, calculated as [(LVEDD–LVDSD)/LVEDD] × 100%) were analysed offline using dedicated software (ProSolv Cardiovascular Analyzer version 3.5; ProSolv, Indianapolis, IN, USA) obtained from M-mode traces. LV mass was calculated as [(IVS+LVPWd+LVEDD)^3^ –LVDSD^3^] × 1.055.

### Measurement of body weight and blood glucose levels

Body weight was monitored every week until mice were sacrificed. Intraperitoneal glucose tolerance test (IPGTT) was carried out after 6 hours of fasting by intraperitoneal injection of 2 g/kg glucose 12 weeks after HFD feeding. Blood glucose levels were determined from tail vein blood by a glucometer (ACCU-CHEK Active, Roche, Mannheim, Germany) before and 15, 30, 60, 90, and 120 min after glucose injection.

### Measurement of fatty acid oxidation (FAO) and oxygen consumption (VO_2_)

To measure FAO, heart muscle tissues were lysed in an ice-cold mitochondria isolation buffer (250 mM sucrose, 10 mM Tris-HCl, and 1 mM EDTA). Lysates were incubated for 2 h with 0.2 mM [^1-14^C] palmitate. ^14^CO_2_ and ^14^C-labelled acid-soluble metabolites were quantified using a liquid scintillation counter. Each cpm value was normalized by the protein content of each lysate. Oxygen consumption rates (VO_2_) of mice were measured using a Columbus Instruments Oxymax System (Columbus, OH, USA). Resting baseline oxygen consumption rates were assessed for at least 1 h.

### Histology and electron microscopic examination

Hearts were immediately isolated for histologic examination and fixed in 4% formaldehyde, dehydrated, embedded in paraffin, and sectioned (4 μm). Sections were stained with haematoxylin and eosin (H&E). To evaluate the degree of interstitial fibrosis, cardiac collagen deposition was assessed by Masson’s trichrome stain (Alfred Pathology, Melbourne, Australia). Images of the LV were obtained using an Olympus light microscope (Tokyo, Japan) at 40x magnification. Collagen stained blue, was measured and analysed using Olympus Image-Pro Plus version 6.0. The percentage of fibrosis observed was calculated by dividing the total area of collagen by the total area of the LV and multiplying by 100%. Data were normalized to a control value of 1 and presented as fold changes.

For transmission electron microscopic examination, heart tissues were dissected, cut into small sections (1 × 1 mm), and immersed in 2.5% glutaraldehyde at 4°C. We calculated the ratio of lipid droplet areas to unit heart muscle area in transmission electron microscopy sections using by Axiovision 4 Imaging/Archiving software (Axiovision 4, Carl Zeiss, Germany). To compare mitochondrial morphology and density among the groups, a blind assessment was performed by a pathologist who randomly selected 10 mitochondria at 3 sites per group and measured long diameters of the cut surfaces.

### Microarray experiment and pathway analysis

Total RNA was purified from heart muscle tissue using RNeasy mini kit (Qiagen, Hilden, Germany). Fragmented biotinylated cRNAs were then generated according to the standard Affymetrix protocol (Affymetrix, Santa Clara, CA, USA). They were hybridized to Affymetrix GeneChip® Mouse Gene 1.0 ST arrays covering 28,853 annotated genes. The fluorescent signal on the array was scanned using a GeneChip® Scanner to produce probe intensities. The log_2_-probe-intensities were normalized and then summarized into log_2_-probeset-intensities using Robust Multiarray Average [13], part of the Expression Console® software (Affymetrix). To identify differentially expressed genes, we performed permutation tests to calculate p values for differential expression. Gene set enrichment analysis (GSEA) [14] was carried out to identify differentially regulated pathways in each group. A total of 1,330 gene sets from public biological pathway databases, or C2 gene sets from mSigDB [14] were analysed for differential regulation. Gene sets or pathways with nominal p values < 0.05 were visualized with network representation by Enrichment Map [15], where two gene sets were connected with an edge if the Jaccard coefficient was greater than > 0.6. The microarray data from this publication have been submitted to the ArrayExpress database (https://www.ebi.ac.uk/arrayexpress/) and assigned the identifier E-MTAB-5329.

### Quantitative real-time polymerase chain reaction (RT-PCR)

cDNA was synthesized using M-MLV reverse transcriptase (Invitrogen, Carlsbad, CA, USA). Quantitative RT-PCR was performed using the ABI 7500 Real-Time PCR System (Applied Biosystems, Foster City, CA, USA), and amplification was achieved using the SYBR Premix Ex Taq polymerase (Takara, Otsu, Japan). Gene-specific primers for *c-fos*, c-Jun-N-terminal kinase (*c-jun*), early growth response 1 (*egr-1*), brain natriuretic peptide (*BNP*), actin a1 skeletal muscle (*Acta1*), sarco/endoplasmic reticulum Ca2+-transport ATPase2a (*Serca2a*), Forkhead box O3 (*FOXO3*), Phosphatase and tensin homolog (*PTEN*), *PPARγ1*, cluster of differentiation 36 (*CD36*, fatty acid translocase), medium-chain acyl-CoA dehydrogenase (*MCAD*), long-chain acyl-CoA dehydrogenase (*LCAD*), very long-chain acyl-CoA dehydrogenase (*VLCAD*), glucose transporter 1(GLUT1), glucose transporter 4 (GLUT4), and pyruvate dehydrogenase kinase 4 (PDK4) (Cosmo Genetech, Seoul, Korea) were designed using Primer Express software (Applied Biosystems). Primer sequences are described in S1 Table. The relative expression of all genes was normalized to both beta actin and 18S RNA, and no differences between normalized levels were found. Therefore, the levels normalized to beta actin are presented.

### Statistical analysis

Results are reported as mean ± standard deviation (SD). Figures are presented as mean ± standard error of the mean (SEM). Statistical analysis was performed with the Mann-Whitney test. Statistical significance was determined at P < 0.05.

## Results

### SHP KO causes cardiac hypertrophy

**Fig 1:**
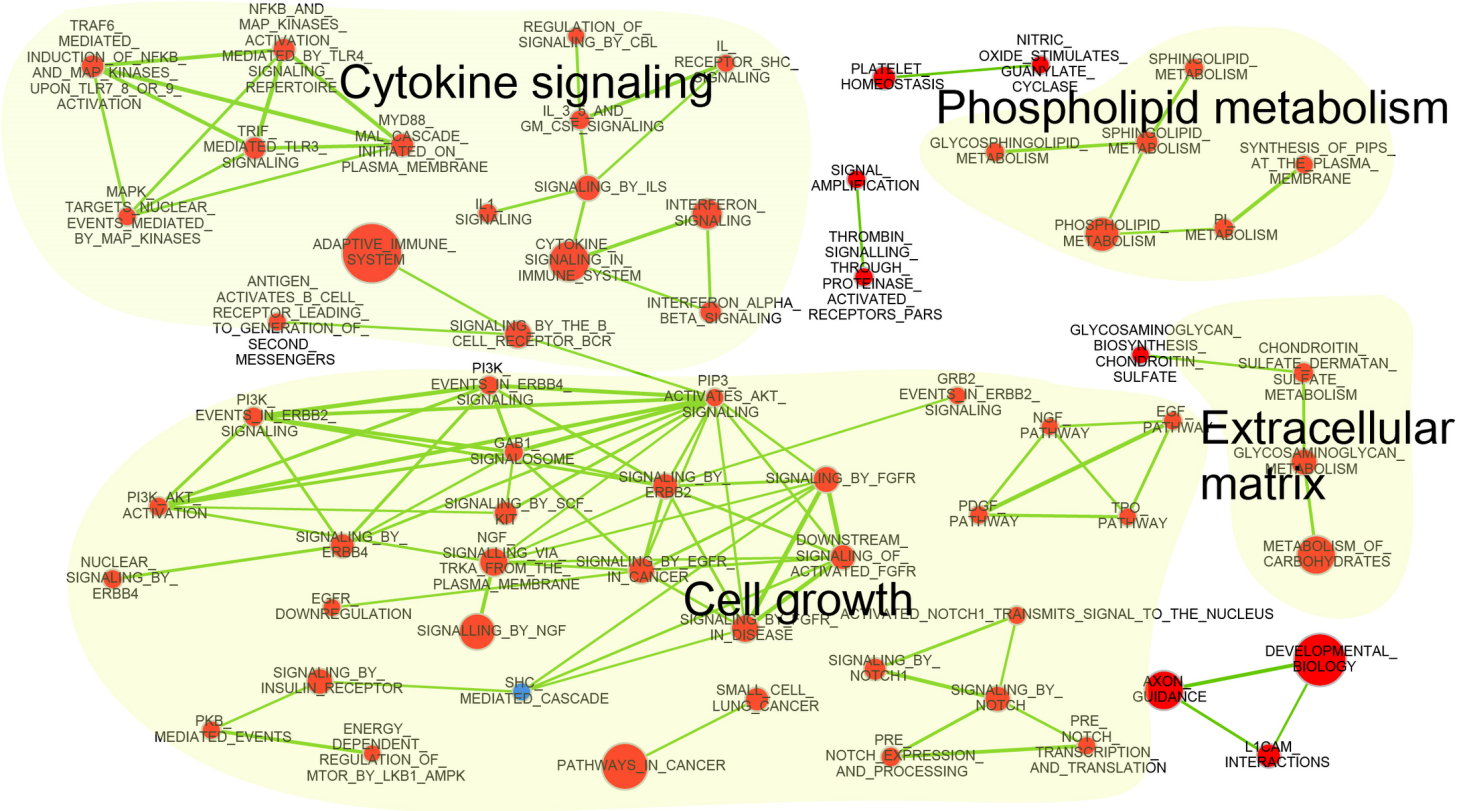
Network of significantly altered biological pathways in the myocardia of SHP KO mice compared to in WT mice. Nodes represent gene sets or pathways, and edges are connected if the two gene sets share a significant number of genes (Jaccard coefficient > 0.6). Gene sets with up-regulated and down-regulated genes in SHP KO mice are coloured red and blue, respectively.

**Fig 2:**
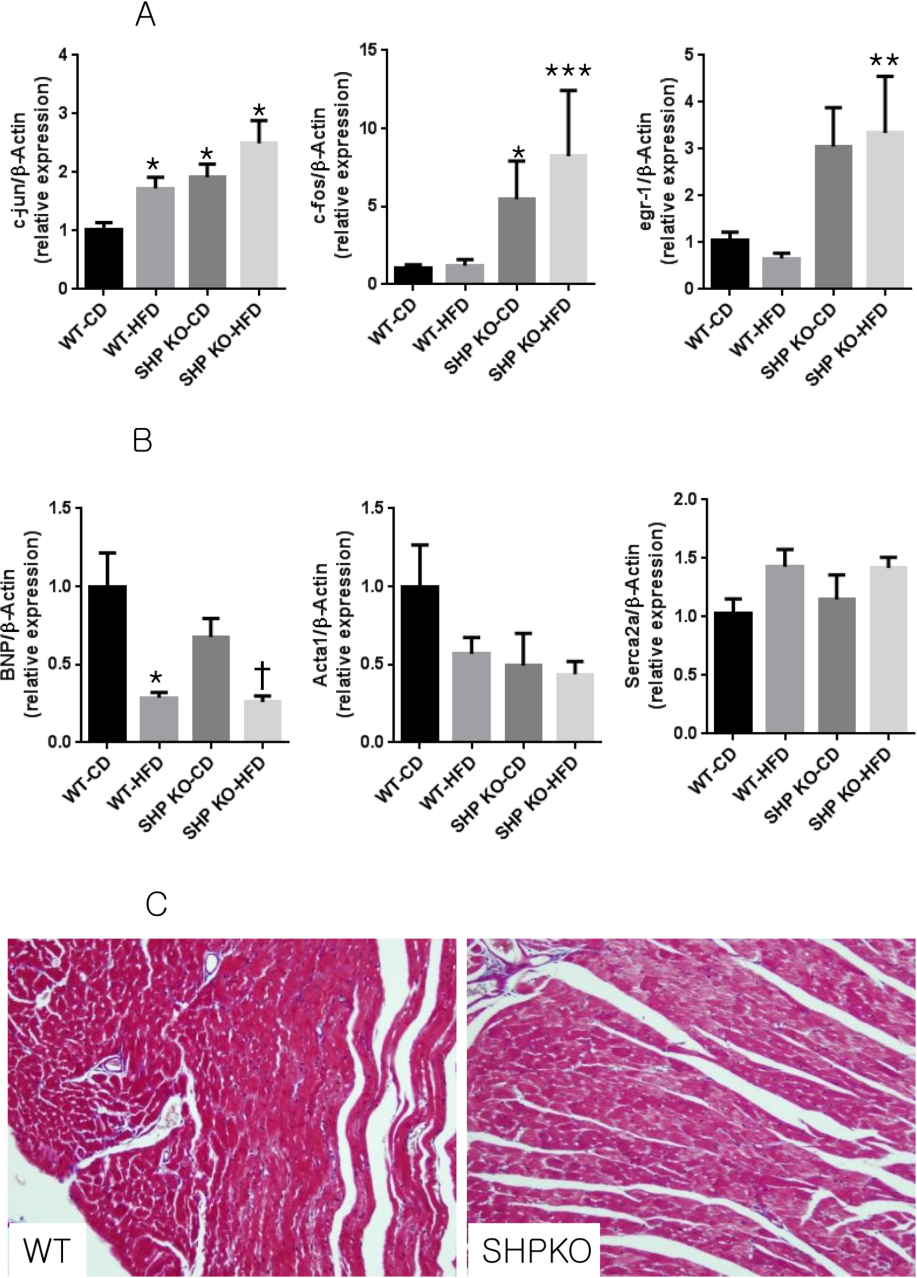
Changes in gene expression induced by SHP deficiency and/or HFD feeding and myocardial histologies. (A) Expression of cell growth-related genes *c-jun*, *c-fos*, and *egr-1*. n = 5–8 per group; * p < 0.05 compared to CD WT mice, ** p < 0.05 compared to HFD WT mice. c-jun, c-Jun-N-terminal kinase; egr-1, early growth response 1. (B) Expression of pathologic hypertrophic markers *BNP*, *Acta1*, and *Serca2a*, normalized to β-actin. n = 5–8 per group; * p < 0.05 compared to CD WT mice, † p < 0.05 compared to CD SHP KO mice. BNP, brain natriuretic peptide; Acta1, actin a1 skeletal muscle; Serca2a, sarco/endoplasmic reticulum Ca^2+^-transport ATPase2a. (C) Myocardial histology of WT and SHP KO mice. Representative myocardial sections of SHP KO mice and WT mice stained with haematoxylin and eosin (H&E). Original magnification, ×200.

Transcriptional profiling with microarrays revealed that pathways involved in cell growth, cytokine signalling, phospholipid metabolism, and extracellular matrix (ECM) were up-regulated in the heart tissue of SHP KO mice compared to in WT mice (Fig 1) under control diets. Interestingly, we found that the PI3K and AKT pathways were also activated in the hearts of SHPKO mice compared to those of WT mice. We used RT-PCR to confirm up-regulation of the proto-oncogenes *c-fos*, *c-jun*, and *egr-1*, the end products of various signal transduction pathways, in the myocardia of SHP KO mice (Fig 2A). Consistent with the increased expression of cell growth-related proto-oncogenes, SHP KO mice exhibited higher LV masses than the WT mice (76.8 ± 20.5 mg vs. 52.8 ± 6.8 mg, P = 0.0093), reflecting cardiac hypertrophy, although there was no difference in ejection fraction or fractional shortening of SHP KO and WT mice (Table 1). However, we did not observe increases in the expression of hypertrophy genes, such as *BNP*, *Acta1*, and *Serca2a*, in the hypertrophied myocardia of SHP KO mice (Fig 2B). In addition, despite the increase in cytokine signalling or ECM-related pathway genes, no significant changes in the infiltration of inflammatory cells or fibrosis were observed in myocardial tissue from SHP KO mice (Fig 2C).

**Table 1:** Morphometric and echocardiographic parameters of wild type and SHP KO mice fed with chow diet or high fat diet for 12 weeks.

	WT-CD (n = 5)	WT-HFD (n = 8)	SHP KO-CD (n = 8)	SHP KO-HFD (n = 8)
Body weight (g)	28.7 ± 1.0	43.5 ± 3.6	31.6 ± 3.4	38.6 ± 3.6†
Heart weight (mg)	132.2 ± 10.7	155.6 ± 18.6	136.6 ± 14.8	134.6 ± 7.0‡
Heart weight/FL (mg/mm)	8.2 ± 0.7	9.6 ± 1.0	8.5 ± 0.1	8.5 ± 0.5‡
LV mass (mg)	52.8 ± 6.8	80.6 ± 22.5*	76.8 ±20.5*	73.9 ± 19.9*
LV mass/FL (mg/mm)	3.3 ± 0.4	5.0 ± 1.4	4.8 ± 1.3	4.2 ± 0.7
IVSd (mm)	0.76 ± 0.09	0.76 ± 0.09	0.88 ± 0.10	0.83 ± 0.10
LVPWd (mm)	0.78 ± 0.11	0.89 ± 0.14	0.86 ± 0.15	0.88 ±0.13
Fractional shortening (%)	62.8 ± 5.5	65.9 ± 5.0	58.7 ± 3.3	63.7 ± 7.6
Ejection fraction (%)	94.6 ± 2.4	95.8 ± 1.7	92.9 ± 1.8	94.6 ± 3.5

Data are means ± SD. FL, femur length; LV, left ventricle; IVSd, Interventricular septal distance; LVPWd, Left ventricular posterior wall distance

* p < 0.05 *vs*. WT-CD mice.

†p < 0.05 *vs*. SHP KO-CD mice

‡ P < 0.05 *vs*. WT-HFD mice

### HFD causes cardiac hypertrophy in WT mice but not in SHP KO mice

**Fig 3:**
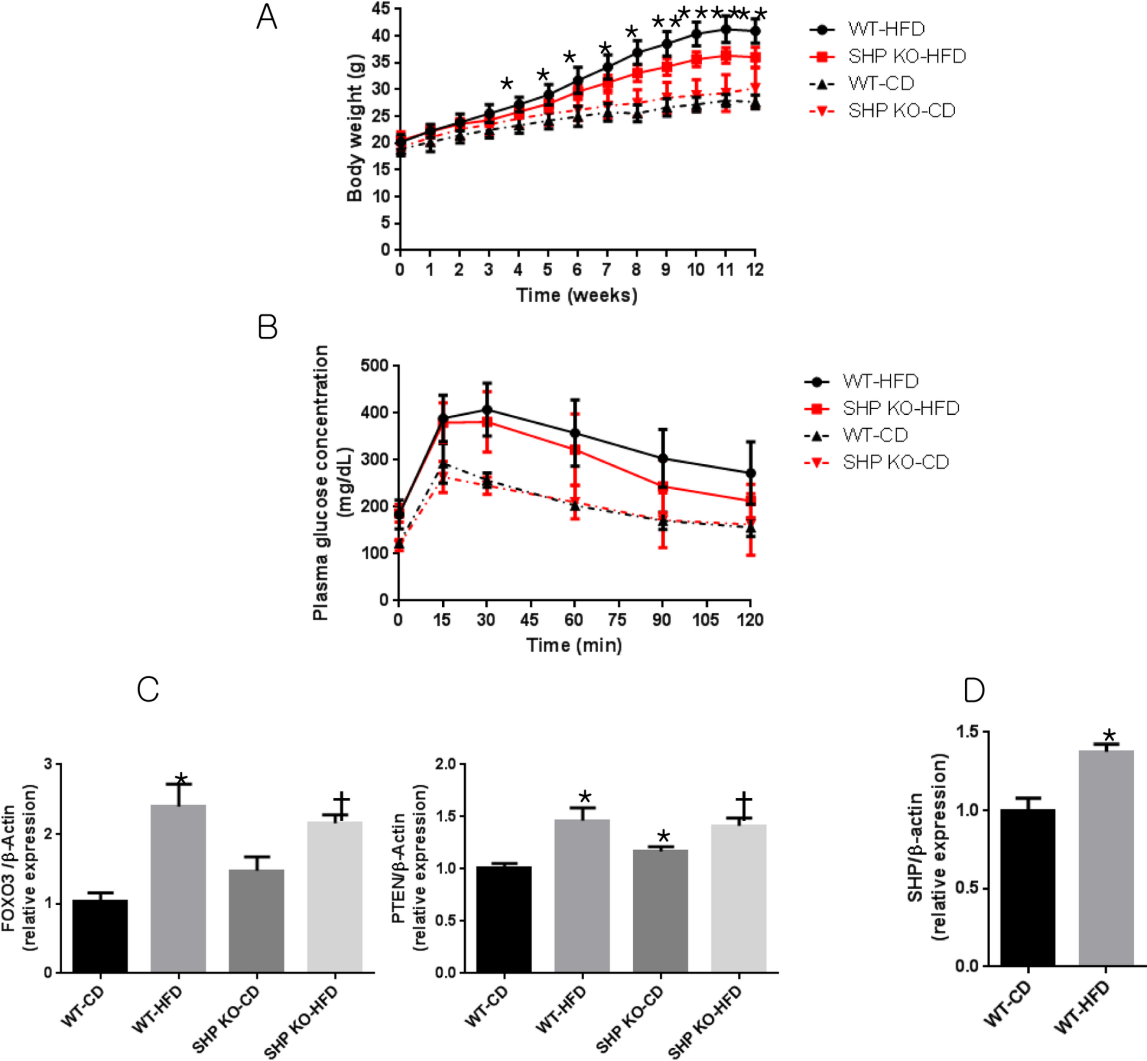
Glucose tolerance and changes in insulin resistance-related genes in the myocardia of WT and SHP KO mice after 12 weeks of HFD feeding. (A) Growth curves in mice. *p < 0.05, ** p < 0.001. (B) Intraperitoneal glucose tolerance tests (IPGTTs) (2 g D-glucose per kg body weight) were performed in 17- to 18-week-old male mice. (C) Relative expression of insulin signalling-related genes *FOXO3* and *PTEN*. n = 5–8 per group; * p < 0.05 compared to CD WT mice, † p < 0.05 compared to CD SHP KO mice. FOXO3, forkhead box O3; PTEN, phosphatase and tensin homolog. (D) Relative expression of *SHP* in WT mice myocardia. n = 5–8 per group; * p < 0.05 compared to CD WT mice.

When SHP KO and WT mice were fed with HFD for 12 weeks, SHP KO mice gained less weight than WT mice without significant changes in blood glucose after 4 weeks (Fig 3A and 3B). HFD WT mice exhibited cardiac hypertrophy when compared to CD WT mice (80.6 ± 22.5 mg vs. 52.8 ± 6.8 mg, P = 0.0227, Table 1), although neither cardiac functional derangements (Table 1) nor increased expression of pathological hypertrophic genes (Fig 2A) were observed. Interestingly, no additional hypertrophy was observed for up to 12 weeks after HFD treatment in SHP KO mice. Transcriptome analysis showed that signal transduction pathways, especially insulin signalling, were down-regulated in HFD WT myocardia (S1 Fig). RT-PCR confirmed that *FOXO3*, which is repressed by insulin signalling, and *PTEN*, a negative regulator of insulin signalling, also increased after HFD (Fig 3C). This suggests that elevated insulin resistance may explain the cardiac hypertrophy observed in HFD WT mice (Table 1). SHP expression was significantly increased in the HFD WT myocardia compared to that in the CD WT myocardia (P = 0.006, Fig 3D).

### SHP KO attenuates HFD-induced lipid accumulation in myocardia

**Fig 4:**
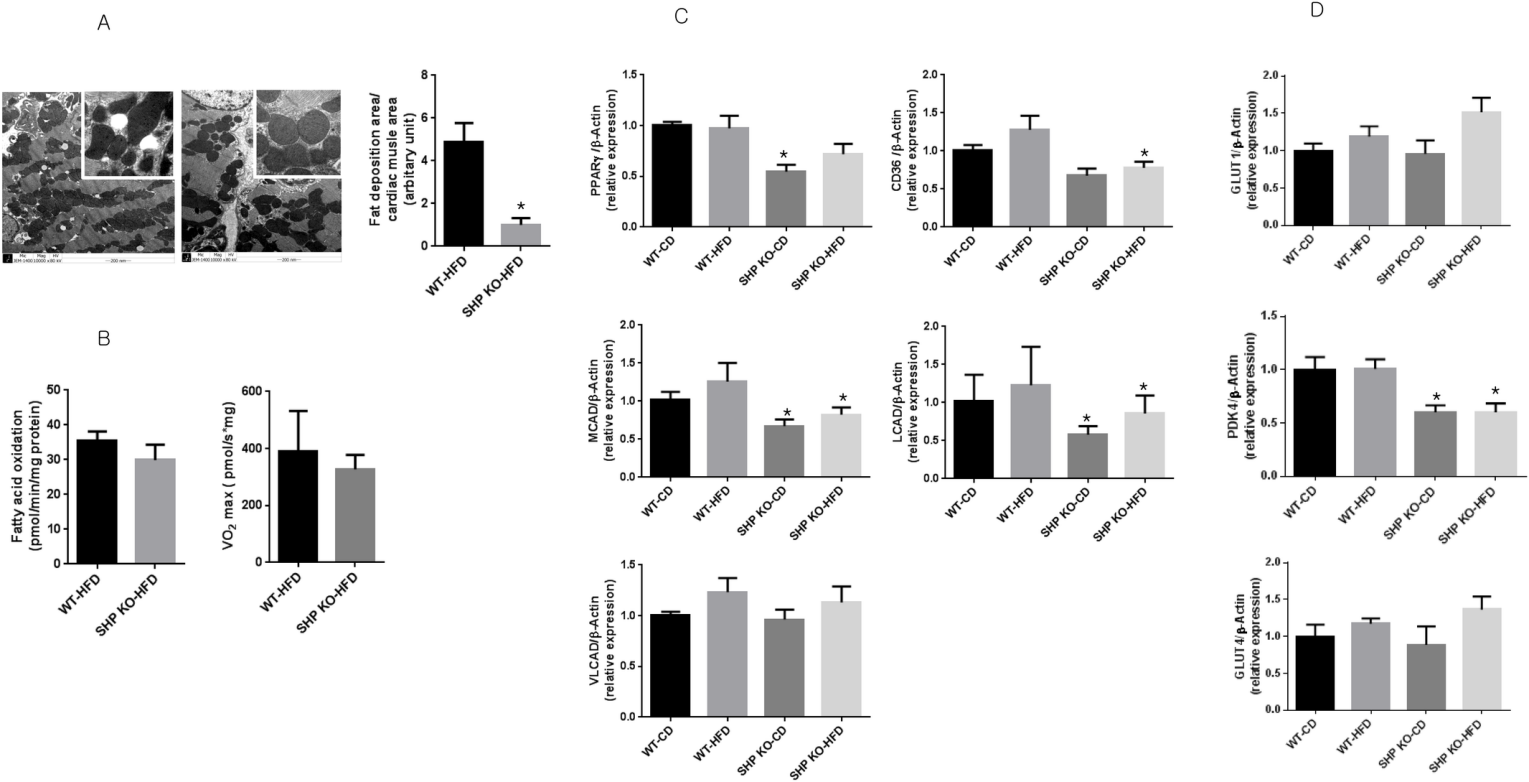
Myocardial lipid accumulation, FAO, and related gene expression in the myocardia of WT and SHP KO mice after 12 weeks of HFD feeding. (A) Lipid droplets in the myocardia of HFD SHP KO and HFD WT mice. Transmission electron microscope images in HFD SHP KO and HFD WT mice and calculated areas of lipid droplets per unit cardiac muscle. Magnifications of large pictures are 10,000× and those of small ones are 500,000×. n = 8 per group; *p < 0.01. (B) Myocardial fatty acid oxidation and VO_2_ max in SHP KO and WT mice fed with HFD. n = 5–8 per group. (C) Expression of PPARγ1 and PPARα target genes *CD36*, *MCAD*, *LCAD*, and *VLCAD* in HFD SHP KO mice. n = 5–8 per group; *p < 0.05 compared to corresponding WT mice. PPARγ1, peroxisome proliferator-activated receptor γ1; CD36, cluster of differentiation; MCAD, medium-chain acyl-CoA dehydrogenase; LCAD, long-chain acyl-CoA dehydrogenase; VLCAD, very long-chain acyl-CoA dehydrogenase. (D) Expression of GLUTs and PDK4 in WT and SHP KO mice. n = 5–8 per group; *p < 0.05 compared to corresponding WT mice. GLUT1, glucose transporter 1; GLUT4, glucose transporter 4; PDK4, pyruvate dehydrogenase kinase 4.

We observed significantly less ectopic lipid accumulation in the myocardial tissue of SHP KO mice (P < 0.01) (Fig 4A), and oxygen consumption and FAO tended to be lower in the myocardia of SHP KO mice, although these trends were not statistically significant (Fig 4B). We found that the long diameters of the mitochondria cut surfaces were not significantly different in the hearts of SHPKO (median of 1.47 μm, range = 0.72–2.06 μm) versus WT (median of 1.22 μm, range = 0.67–2.11 μm) mice. Further, there were no significant differences in mitochondrial morphology and density (S2 Fig), and no abnormal material accumulation was observed. Next, we investigated transcriptional changes in the hearts of SHP KO mice compared to in WT mice after HFD. Table 2 shows the list of pathways significantly down-regulated in the myocardia of SHP KO mice. PPARγ1 and PPARα target genes were down-regulated in SHP KO myocardia after HFD, suggesting that the decrease in lipogenesis after SHP KO may explain the decrease in myocardial lipid accumulation. We confirmed the reduced expression of the PPARγ1 and PPARα target genes *CD36*, *MCAD*, *LCAD*, and *VLCAD* in SHP KO mice (Fig 4C). Moreover, SHP KO mice exhibited less elevation in serum free fatty acid compared with WT mice after HFD (859.4 ± 166.3 mg/dL vs. 720.0 ± 62.0 mg/dL, P = 0.005). To evaluate substrate preference between free fatty acid and glucose in myocardia, the expression of GLUTs and PDK4 were examined. We found that the transcriptional level of PDK4 was profoundly decreased in the hearts of SHP KO mice compared to that of WT mice (P < 0.001, Fig 4D), and we found marginally increased expression of GLUT1 in the hearts of HFD SHP KO mice compared to that of CD SHP KO mice (Fig 4D). However, the expression of GLUT1 and GLUT4 in the heart was not different between WT and SHP KO mice.

**Table 2:** The list of gene sets down-regulated in heart tissue from SHP KO HFD mice compared to that from WT HFD mice (nominal P values < 0.001).

Name	Description	Size	ES	NES
INTRINSIC PATHWAY (BIOCARTA)	Intrinsic prothrombin activation pathway	22	-0.88	-1.15
FORMATION OF FIBRIN CLOT CLOTTING CASCADE (REACTOME)	Genes involved in formation of fibrin clot (clotting cascade)	29	-0.85	-1.25
INTRINSIC PATHWAY (REACTOME)	Intrinsic prothrombin activation pathway	15	-0.83	-1.39
PHENYLALANINE METABOLISM (KEGG)	Phenylalanine metabolism	16	-0.78	-1.20
BIOSYNTHESIS OF UNSATURATED FATTY ACIDS (KEGG)	Biosynthesis of unsaturated fatty acids	18	-0.67	-1.05
SELENOAMINO ACID METABOLISM (KEGG)	Selenoamino acid metabolism	24	-0.65	-1.20
EPHRINB REV PATHWAY (PID)	Ephrin B reverse signaling	29	-0.63	-1.47
PPARA PATHWAY (BIOCARTA)	Mechanism of gene regulation by peroxisome proliferators via PPARa(alpha)	54	-0.61	-1.63
PROPANOATE METABOLISM (KEGG)	Propanoate metabolism	29	-0.61	-1.41
CITRIC ACID CYCLE TCA CYCLE (REACTOME)	Genes involved in citric acid cycle (TCA cycle)	18	-0.58	-1.41
GLYPICAN 1PATHWAY (PID)	Glypican 1 network	24	-0.52	-1.38
AMINO ACID SYNTHESIS AND INTERCONVERSION TRANSAMINATION (REACTOME)	Genes involved in amino acid synthesis and interconversion (transamination)	16	-0.52	-1.35
PPARG 01	Genes having at least one occurrence of the transcription factor binding site V$PPARG_01 (v7.4 TRANSFAC) in the regions spanning up to 4 kb around their transcription start sites	33	-0.48	-1.42
PYRUVATE METABOLISM AND CITRIC ACID TCA CYCLE (REACTOME)	Genes involved in pyruvate metabolism and citric Acid (TCA) cycle	37	-0.48	-1.68
GGTAACC, MIR-409-5P	Genes having at least one occurrence of the motif GGTAACC in their 3' untranslated region. The motif represents putative target of human mature miRNA hsa-miR-409-5p (v7.1 miRBase)	30	-0.47	-1.53
METABOLISM OF VITAMINS AND COFACTORS (REACTOME)	Genes involved in metabolism of vitamins and cofactors	46	-0.46	-1.24
GLUTAMATE NEUROTRANSMITTER RELEASE CYCLE (REACTOME)	Genes involved in glutamate neurotransmitter release cycle	15	-0.44	-1.29
PPARA 01	Genes having at least one occurrence of the transcription factor binding site V$PPARA_01 (v7.4 TRANSFAC) in the regions spanning up to 4 kb around their transcription start sites	32	-0.42	-1.37
INTEGRIN CELL SURFACE INTERACTIONS (REACTOME)	Genes involved in integrin cell surface interactions	75	-0.41	-1.42
PTEN PATHWAY SA	PTEN is a tumor suppressor that dephosphorylates the lipid messenger phosphatidylinositol triphosphate	17	-0.40	-1.40
RYAAAKNNNNNNTTGW UNKNOWN	Genes having at least one occurrence of the highly conserved motif M151 RYAAAKNNNNNNTTGW in the region spanning up to 4 kb around their transcription start sites. The motif does not match any known transcription factor binding site	73	-0.35	-1.31
NECTIN PATHWAY (PID)	Nectin adhesion pathway	29	-0.35	-1.36
FXR Q3	Genes having at least one occurrence of the transcription factor binding site V$FXR_Q3 (v7.4 TRANSFAC) in the regions spanning up to 4 kb around their transcription start sites	100	-0.32	-1.16
E2F Q2	Genes having at least one occurrence of the transcription factor binding site V$E2F_Q2 (v7.4 TRANSFAC) in the regions spanning up to 4 kb around their transcription start sites	147	-0.31	-1.45
GGCACTT, MIR-519E	Genes having at least one occurrence of the motif GGCACTT in their 3' untranslated region. The motif represents putative target (i.e., seed match) of human mature miRNA hsa-miR-519e (v7.1 miRBase)	109	-0.30	-1.52
COUP DR1 Q6	Genes having at least one occurrence of the transcription factor binding site V$COUP_DR1_Q6 (v7.4 TRANSFAC) in the regions spanning up to 4 kb around their transcription start sites	208	-0.30	-1.34
TGFB PATHWAY (BIOCARTA)	TGF beta signaling pathway	18	-0.30	-1.15
MAZ Q6	Genes having at least one occurrence of the transcription factor binding site V$MAZ_Q6 (v7.4 TRANSFAC) in the regions spanning up to 4 kb around their transcription start sites	169	-0.29	-1.39
DBP Q6	Genes having at least one occurrence of the transcription factor binding site V$DBP_Q6 (v7.4 TRANSFAC) in the regions spanning up to 4 kb around their transcription start sites	212	-0.26	-1.26
AUTODEGRADATION OF CDH1 BY CDH1 APC C (REACTOME)	Genes involved in autodegradation of Cdh1 by Cdh1:APC/C	51	-0.25	-1.37
GCGSCMNTTT UNKNOWN	Genes having at least one occurrence of the highly conserved motif M164 GCGSCMNTTT in the region spanning up to 4 kb around their transcription start sites. The motif does not match any known transcription factor binding site	55	-0.25	-1.21
USF2 Q6	Genes having at least one occurrence of the transcription factor binding site V$USF2_Q6 (v7.4 TRANSFAC) in the regions spanning up to 4 kb around their transcription start sites	218	-0.23	-1.13
GATA1 02	Genes having at least one occurrence of the transcription factor binding site V$GATA1_02 (v7.4 TRANSFAC) in the regions spanning up to 4 kb around their transcription start sites	210	-0.22	-1.14
NFMUE1 Q6	Genes having at least one occurrence of the transcription factor binding site V$NFMUE1_Q6 (v7.4 TRANSFAC) in the regions spanning up to 4 kb around their transcription start sites	204	-0.19	-1.43
MYCMAX 03	Genes having at least one occurrence of the transcription factor binding site V$MYCMAX_03 (v7.4 TRANSFAC) in the regions spanning up to 4 kb around their transcription start sites	212	-0.19	-1.11

**ES**, enrichment score; **NES**, normalized enrichment score; **BIOCARTA**, BioCarta pathway database; **REACTOME**, Reactome pathway database; **KEGG**, KEGG pathway database; **PID**, Pathway Interaction Database; **SA**, Sigma-Aldrich pathway database; **TRANSFAC**, TRANSFAC database; **miRBase**, microRNA database.

## Discussion

In the present study, SHP deficiency induced cardiac hypertrophy, and consistent with this, up-regulation of genes involved in cell growth, cytokine signalling, phospholipid metabolism, and ECM in the heart tissue of SHP KO mice. These effects were associated with a decrease in plasma free fatty acid levels and reduced deposition of fat in the myocardia of HFD SHP KO mice. SHP deficiency did not increase O_2_ consumption or FAO in myocardia. PPARγ1 and PPARα target genes were down-regulated in the myocardia of HFD SHP KO mice, and expression levels of several key genes involving fatty acid uptake, oxidation, and synthesis were decreased in the hearts of HFD SHP KO mice compared with levels in HFD WT mice.

In the liver, sustained SHP expression results in depletion of the hepatic bile acid pool and triglyceride accumulation. In contrast, SHP deficiency is associated with reduced hepatic cholesterol, triglyceride, and free fatty acid levels compared to WT levels [7, 16]. Moreover, SHP-deficient mice exhibit increased fatty acid oxidation and decreased lipogenesis; thus, they are protected from diet-induced hepatic steatosis by very low expression of PPARγ2 [7]. These findings in the liver are in partial agreement with those in the myocardium in terms of the reduced lipid deposition. It could be possible through down-regulated expressions of PPARγ target genes. Unlike in the liver, in the myocardia of HFD SHP KO mice, FAO was not increased but the expression levels of *PPARγ1*, *CD36*, *MCAD*, and *LCAD*, key genes involved in fatty acid uptake and oxidation, were significantly decreased according to the present study. These findings could reflect the down-regulation of PPARα target genes. In agreement with our findings, PPARα-deficient mice were previously reported to exhibit age-associated cardiac fibrosis, diminished rates of FAO, and a lack of cellular fasting responses [17, 18]. MCAD is pivotal in catalyzing mitochondrial FAO and decreased MCAD expression was reported in pressure overload *in vivo* models [19, 20]. Consistent with these findings, the expression of MCAD was also reduced in the hearts of SHP KO mice in the present study. While a decrease in FAO in the myocardium can induce cardiac fat accumulation, reduced lipid accumulation was observed in the present study. This may be the result of an indirect effect of low levels of plasma free fatty acids from the liver or adipose tissue due to down-regulation of PPARγ target gene expression. In agreement with these findings, SHP overexpression in cardiomyocytes has been reported to induce lipid accumulation, which was accompanied with increased CD36 and PPARγ expression [12]. We also observed that SHP mRNA levels are induced in the hearts of WT mice fed with HFD, as shown in the previous study. Nam *et al*., however, reported that metformin induces SHP in an AMPK-independent manner, and that SHP is a novel anti-hypertrophic regulator mediating the anti-hypertrophic role of metformin in the heart [11]. Admittedly, these findings are in contrast with ours in the present study. Regarding cardiac hypertrophy, SHP has a beneficial role. As we reported in the current study, SHP loss is associated with cardiac hypertrophy. However, from the perspective of lipid metabolism, we also found that SHP loss is beneficial with reduced ectopic lipid accumulation in the heart after HFD. Therefore, whether SHP plays a beneficial role is dependent on the condition—cardiac hypertrophy or lipid metabolism.

The process of selecting fatty acid or glucose for fuel in cells during the fed-fast cycle is largely controlled by the pyruvate dehydrogenase complex, which is regulated by pyruvate dehydrogenase kinase (PDK) and pyruvate dehydrogenase phosphatases [21]. Especially, a role of PDK4 in cardiac hypertrophy and dilated cardiomyopathy has been reported [22–24]. In the present study, we observed a profound decrease in the expression of PDK4 in the hearts of SHP KO mice. We propose that the reduction in plasma free fatty acids caused by decreased expression of PPARγ1 or PPARα could reduce the expression of PDK4.

SHP functions predominantly as a transcriptional repressor of gene expression by directly binding to a variety of nuclear receptors [1]. In our microarray experiment, various signal transduction pathway genes were up-regulated by SHP KO. The end products of various signal transduction pathways are proto-oncogenes, such as *c-fos*, *c-jun*, and *egr-1*, which were significantly up-regulated in the myocardia of SHP KO mice. The de-repression of transcriptional regulators by SHP deletion may have caused cardiac hypertrophy by activating these proto-oncogenes. Nam *et al*. reported that the GATA-6 signalling pathway is responsible for cardiac hypertrophy following SHP deletion [11]. In the current study, microarray analysis also confirmed that expression of GATA-6 target genes was increased by SHP knockout (nominal p < 0.01). However, our genome-wide transcriptional profiling study further suggests that the global increase in multiple signalling pathway genes and proto-oncogenes induced by SHP knockout may explain the cardiac hypertrophy observed in SHP KO mice.

It has been reported that activation of the PI3K and AKT pathways is important for cardiomyopathy, especially when they are chronically activated [25]. In the present study, we found that the PI3K and AKT pathways were activated in the hearts of SHP KO mice compared to those of WT mice, suggesting a chronic activation of these pathways in the hearts of SHP KO mice. This finding is different from that found from decreased acute activation of the PI3K and AKT pathways in insulin resistance induced by a HFD or obesity.

Whereas cardiac hypertrophy by SHP deletion is associated with increases in multiple signalling pathways, the increase in the LV mass observed in HFD mice seems to result from the development of insulin resistance. A previous study reported that FOXO is activated by HFD and that diabetic cardiomyopathy, characterized by cardiac hypertrophy, is caused by insulin resistance due to FOXO activation [26]. Although we failed to observe cardiac dysfunction over 12 weeks of HFD, insulin resistance did develop, as demonstrated by increases in *FOXO3* and *PTEN* levels. A longer treatment period (i.e. more than 6 months) or additional stimuli such as a transverse aortic constriction will be needed to induce a more pronounced cardiac phenotype [27–29].

A limitation of this study is that we cannot distinguish whether the observed metabolic change in the myocardium of SHP-deficient mice is the result of a direct heart-specific effect of SHP deletion or from a systemic correction of body metabolism, as we used a whole-body SHP knockout model. A future study using cardiac-specific KO of SHP is warranted to clarify the direct role of SHP deletion in the heart.

In conclusion, SHP-deficient mouse hearts exhibited hypertrophy but reduced ectopic lipid accumulation after HFD, likely resulting from a decrease in lipogenesis regulated by PPARγ1.

## Supporting information

S1 Fig Network of significantly altered biological pathways in the myocardia of SHP KO mice compared to in WT mice after 12 weeks of HFD feeding. Nodes represent gene sets or pathways, and edges are connected if the two gene sets share a significant number of genes (Jaccard coefficient > 0.6). Gene sets with up-regulated and down-regulated genes in SHP KO mice are coloured red and blue, respectively.
(TIF)

S2 Fig Representative mitochondrial morphologies using electron microscopy (×25,000) in WT mice and SHP KO mice. There were no significant differences in mitochondrial morphology and density between WT mice and SHP KO mice.
(TIF)

S1 Table Primer sequences used in quantitative real-time polymerase chain reaction. (DOCX)

## References

[1] Zhang Y, Hagedorn CH, Wang L. Role of nuclear receptor SHP in metabolism and cancer. Biochimica et biophysica acta. 2011;1812(8):893–908. Epub 2010/10/26. doi: 10.1016/j.bbadis.2010.10.006 ; PubMed Central PMCID: PMC3043166. 20970497 10.1016/j.bbadis.2010.10.006 PMC3043166

[2] Chanda D, Park JH, Choi HS. Molecular basis of endocrine regulation by orphan nuclear receptor Small Heterodimer Partner. Endocrine journal. 2008;55(2):253–68. Epub 2007/11/07. . 17984569 10.1507/endocrj.k07e-103

[3] Wang L, Liu J, Saha P, Huang J, Chan L, Spiegelman B, et al The orphan nuclear receptor SHP regulates PGC-1alpha expression and energy production in brown adipocytes. Cell metabolism. 2005;2(4):227–38. Epub 2005/10/11. doi: 10.1016/j.cmet.2005.08.010 . 16213225 10.1016/j.cmet.2005.08.010

[4] Boulias K, Katrakili N, Bamberg K, Underhill P, Greenfield A, Talianidis I. Regulation of hepatic metabolic pathways by the orphan nuclear receptor SHP. The EMBO journal. 2005;24(14):2624–33. Epub 2005/06/24. doi: 10.1038/sj.emboj.7600728 ; PubMed Central PMCID: PMC1176456. 15973435 10.1038/sj.emboj.7600728 PMC1176456

[5] Huang J, Iqbal J, Saha PK, Liu J, Chan L, Hussain MM, et al Molecular characterization of the role of orphan receptor small heterodimer partner in development of fatty liver. Hepatology. 2007;46(1):147–57. Epub 2007/05/26. doi: 10.1002/hep.21632 . 17526026 10.1002/hep.21632

[6] Hartman HB, Lai K, Evans MJ. Loss of small heterodimer partner expression in the liver protects against dyslipidemia. Journal of lipid research. 2009;50(2):193–203. Epub 2008/09/30. doi: 10.1194/jlr.M800323-JLR200 . 18820241 10.1194/jlr.M800323-JLR200

[7] Park YJ, Kim SC, Kim J, Anakk S, Lee JM, Tseng HT, et al Dissociation of diabetes and obesity in mice lacking orphan nuclear receptor small heterodimer partner. Journal of lipid research. 2011;52(12):2234–44. Epub 2011/09/29. doi: 10.1194/jlr.M016048 ; PubMed Central PMCID: PMC3220290. 21949050 10.1194/jlr.M016048 PMC3220290

[8] Nishizawa H, Yamagata K, Shimomura I, Takahashi M, Kuriyama H, Kishida K, et al Small heterodimer partner, an orphan nuclear receptor, augments peroxisome proliferator-activated receptor gamma transactivation. The Journal of biological chemistry. 2002;277(2):1586–92. Epub 2001/11/07. doi: 10.1074/jbc.M104301200 . 11696534 10.1074/jbc.M104301200

[9] Saddik M, Lopaschuk GD. Myocardial triglyceride turnover and contribution to energy substrate utilization in isolated working rat hearts. The Journal of biological chemistry. 1991;266(13):8162–70. Epub 1991/05/05. . 1902472

[10] Ingwall JS. Energy metabolism in heart failure and remodelling. Cardiovascular research. 2009;81(3):412–9. Epub 2008/11/07. doi: 10.1093/cvr/cvn301 ; PubMed Central PMCID: PMC2639129. 18987051 10.1093/cvr/cvn301 PMC2639129

[11] Nam YS, Kim Y, Joung H, Kwon DH, Choe N, Min HK, et al Small heterodimer partner blocks cardiac hypertrophy by interfering with GATA6 signaling. Circulation research. 2014;115(5):493–503. Epub 2014/07/13. doi: 10.1161/CIRCRESAHA.115.304388 . 25015078 10.1161/CIRCRESAHA.115.304388

[12] Rodriguez-Calvo R, Chanda D, Oligschlaeger Y, Miglianico M, Coumans WA, Barroso E, et al Small heterodimer partner (SHP) contributes to insulin resistance in cardiomyocytes. Biochimica et biophysica acta. 2017;1862(5):541–51. Epub 2017/02/20. doi: 10.1016/j.bbalip.2017.02.006 . 28214558 10.1016/j.bbalip.2017.02.006

[13] Irizarry RA, Bolstad BM, Collin F, Cope LM, Hobbs B, Speed TP. Summaries of Affymetrix GeneChip probe level data. Nucleic acids research. 2003;31(4):e15 Epub 2003/02/13. ; PubMed Central PMCID: PMC150247. 12582260 10.1093/nar/gng015 PMC150247

[14] Subramanian A, Tamayo P, Mootha VK, Mukherjee S, Ebert BL, Gillette MA, et al Gene set enrichment analysis: a knowledge-based approach for interpreting genome-wide expression profiles. Proceedings of the National Academy of Sciences of the United States of America. 2005;102(43):15545–50. Epub 2005/10/04. doi: 10.1073/pnas.0506580102 ; PubMed Central PMCID: PMC1239896. 16199517 10.1073/pnas.0506580102 PMC1239896

[15] Merico D, Isserlin R, Stueker O, Emili A, Bader GD. Enrichment map: a network-based method for gene-set enrichment visualization and interpretation. PloS one. 2010;5(11):e13984 Epub 2010/11/19. doi: 10.1371/journal.pone.0013984 ; PubMed Central PMCID: PMC2981572. 21085593 10.1371/journal.pone.0013984 PMC2981572

[16] Wang L, Han Y, Kim CS, Lee YK, Moore DD. Resistance of SHP-null mice to bile acid-induced liver damage. The Journal of biological chemistry. 2003;278(45):44475–81. Epub 2003/08/23. doi: 10.1074/jbc.M305258200 . 12933814 10.1074/jbc.M305258200

[17] Finck BN. The PPAR regulatory system in cardiac physiology and disease. Cardiovascular research. 2007;73(2):269–77. Epub 2006/10/03. doi: 10.1016/j.cardiores.2006.08.023 . 17010956 10.1016/j.cardiores.2006.08.023

[18] Leone TC, Weinheimer CJ, Kelly DP. A critical role for the peroxisome proliferator-activated receptor alpha (PPARalpha) in the cellular fasting response: the PPARalpha-null mouse as a model of fatty acid oxidation disorders. Proceedings of the National Academy of Sciences of the United States of America. 1999;96(13):7473–8. Epub 1999/06/23. ; PubMed Central PMCID: PMC22110. 10377439 10.1073/pnas.96.13.7473 PMC22110

[19] Sack MN, Rader TA, Park S, Bastin J, McCune SA, Kelly DP. Fatty acid oxidation enzyme gene expression is downregulated in the failing heart. Circulation. 1996;94(11):2837–42. Epub 1996/12/01. . 8941110 10.1161/01.cir.94.11.2837

[20] Osorio JC, Stanley WC, Linke A, Castellari M, Diep QN, Panchal AR, et al Impaired myocardial fatty acid oxidation and reduced protein expression of retinoid X receptor-alpha in pacing-induced heart failure. Circulation. 2002;106(5):606–12. Epub 2002/07/31. . 12147544 10.1161/01.cir.0000023531.22727.c1

[21] Jeong JY, Jeoung NH, Park KG, Lee IK. Transcriptional regulation of pyruvate dehydrogenase kinase. Diabetes & metabolism journal. 2012;36(5):328–35. Epub 2012/11/07. doi: 10.4093/dmj.2012.36.5.328 ; PubMed Central PMCID: PMC3486978. 23130316 10.4093/dmj.2012.36.5.328 PMC3486978

[22] Lee IK. The role of pyruvate dehydrogenase kinase in diabetes and obesity. Diabetes & metabolism journal. 2014;38(3):181–6. Epub 2014/07/09. doi: 10.4093/dmj.2014.38.3.181 ; PubMed Central PMCID: PMC4083023. 25003070 10.4093/dmj.2014.38.3.181 PMC4083023

[23] Kong SW, Bodyak N, Yue P, Liu Z, Brown J, Izumo S, et al Genetic expression profiles during physiological and pathological cardiac hypertrophy and heart failure in rats. Physiological genomics. 2005;21(1):34–42. Epub 2004/12/30. doi: 10.1152/physiolgenomics.00226.2004 . 15623566 10.1152/physiolgenomics.00226.2004

[24] Arikawa E, Ma RC, Isshiki K, Luptak I, He Z, Yasuda Y, et al Effects of insulin replacements, inhibitors of angiotensin, and PKCbeta's actions to normalize cardiac gene expression and fuel metabolism in diabetic rats. Diabetes. 2007;56(5):1410–20. Epub 2007/03/17. doi: 10.2337/db06-0655 . 17363743 10.2337/db06-0655

[25] Matsui T, Li L, Wu JC, Cook SA, Nagoshi T, Picard MH, et al Phenotypic spectrum caused by transgenic overexpression of activated Akt in the heart. The Journal of biological chemistry. 2002;277(25):22896–901. Epub 2002/04/12. doi: 10.1074/jbc.M200347200 . 11943770 10.1074/jbc.M200347200

[26] Battiprolu PK, Hojayev B, Jiang N, Wang ZV, Luo X, Iglewski M, et al Metabolic stress-induced activation of FoxO1 triggers diabetic cardiomyopathy in mice. The Journal of clinical investigation. 2012;122(3):1109–18. Epub 2012/02/14. doi: 10.1172/JCI60329 ; PubMed Central PMCID: PMC3287230. 22326951 10.1172/JCI60329 PMC3287230

[27] Fang CX, Dong F, Thomas DP, Ma H, He L, Ren J. Hypertrophic cardiomyopathy in high-fat diet-induced obesity: role of suppression of forkhead transcription factor and atrophy gene transcription. American journal of physiology Heart and circulatory physiology. 2008;295(3):H1206–H15. Epub 2008/07/22. doi: 10.1152/ajpheart.00319.2008 ; PubMed Central PMCID: PMC2544483. 18641278 10.1152/ajpheart.00319.2008 PMC2544483

[28] Wang Z, Li L, Zhao H, Peng S, Zuo Z. Chronic high fat diet induces cardiac hypertrophy and fibrosis in mice. Metabolism: clinical and experimental. 2015;64(8):917–25. Epub 2015/05/20. doi: 10.1016/j.metabol.2015.04.010 ; PubMed Central PMCID: PMC4461501. 25982698 10.1016/j.metabol.2015.04.010 PMC4461501

[29] Calligaris SD, Lecanda M, Solis F, Ezquer M, Gutierrez J, Brandan E, et al Mice long-term high-fat diet feeding recapitulates human cardiovascular alterations: an animal model to study the early phases of diabetic cardiomyopathy. PloS one. 2013;8(4):e60931 Epub 2013/04/18. doi: 10.1371/journal.pone.0060931 ; PubMed Central PMCID: PMC3623942. 23593350 10.1371/journal.pone.0060931 PMC3623942

