# Supplementary figures and images for: Small heterodimer partner (SHP) deficiency protects myocardia from lipid accumulation in high fat diet-fed mice
Source: PLoS One. 2017 Oct 10;12(10):e0186021. doi: 10.1371/journal.pone.0186021 (PMC5634594; doi:10.1371/journal.pone.0186021)

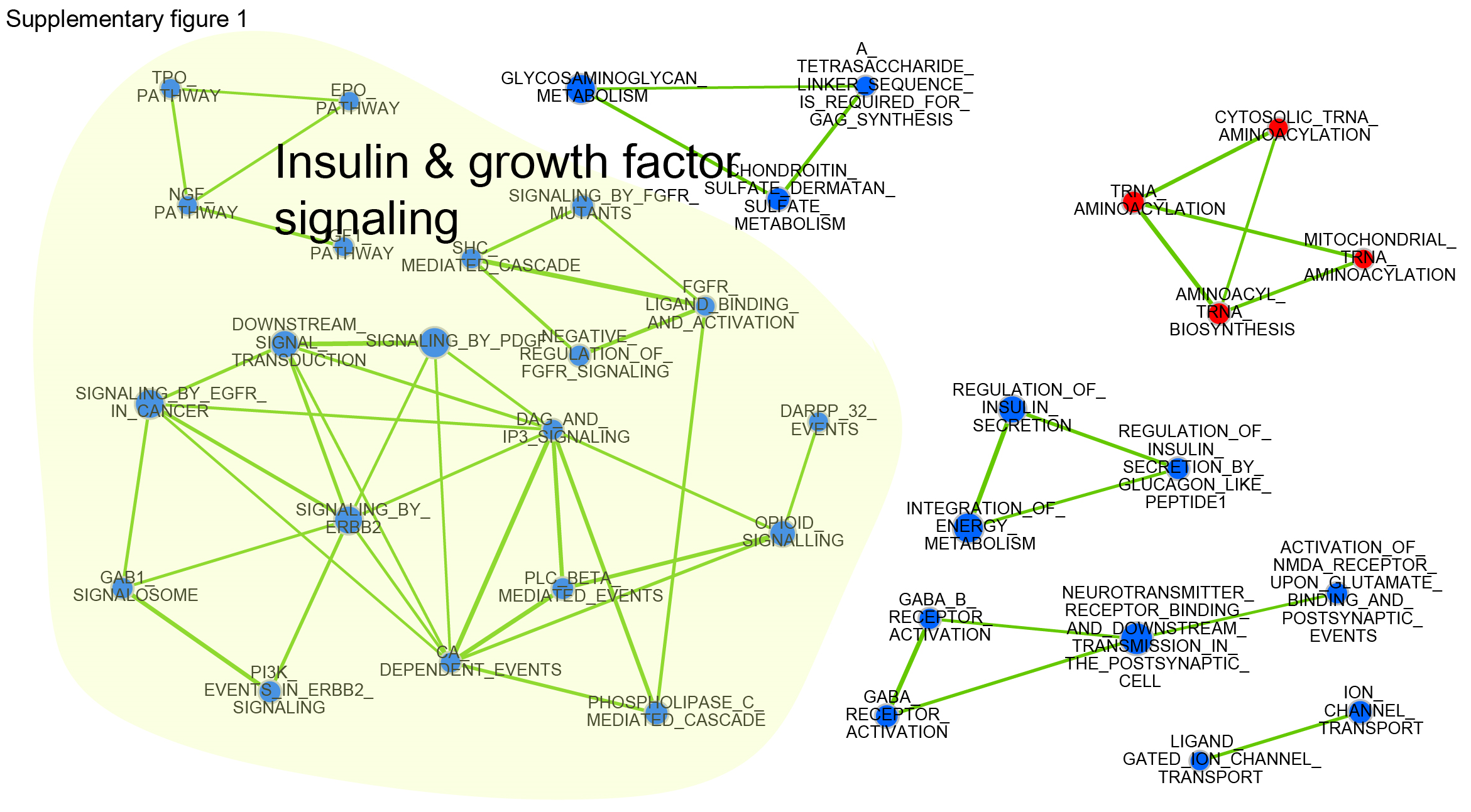

Supplement: S1 Fig — Nodes represent gene sets or pathways, and edges are connected if the two gene sets share a significant number of genes (Jaccard coefficient > 0.6). Gene sets with up-regulated and down-regulated genes in SHP KO mice are coloured red and blue, respectively. (TIF) [file pone.0186021.s001.tif]

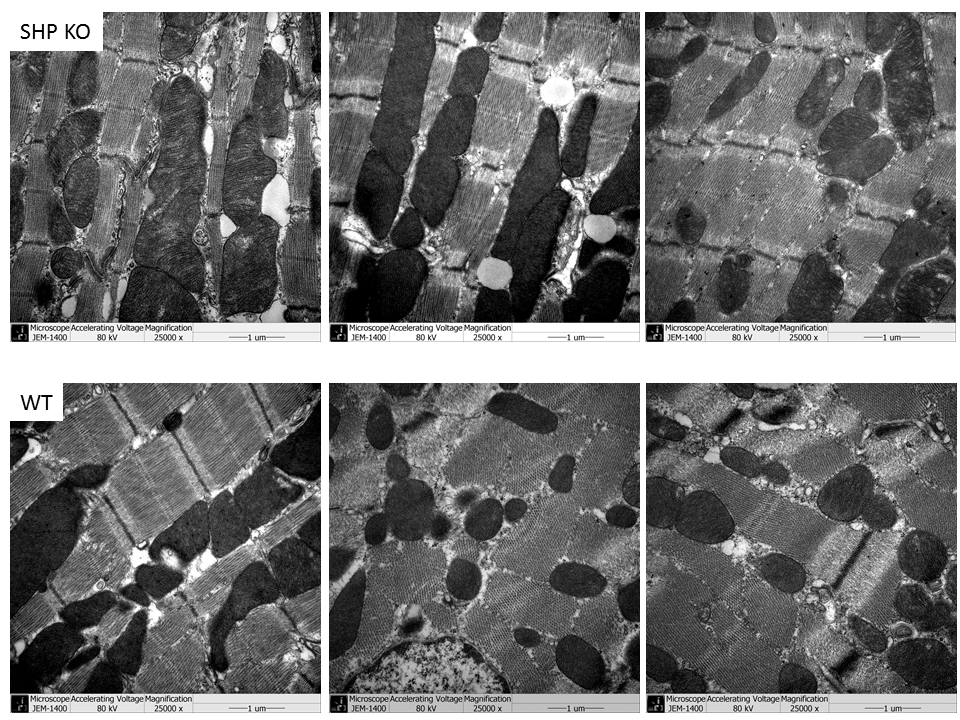

Supplement: S2 Fig — There were no significant differences in mitochondrial morphology and density between WT mice and SHP KO mice. (TIF) [file pone.0186021.s002.tif]
