# Supplementary material for: Small heterodimer partner (SHP) deficiency protects myocardia from lipid accumulation in high fat diet-fed mice
Source: PLoS One. 2017 Oct 10;12(10):e0186021. doi: 10.1371/journal.pone.0186021 (PMC5634594; doi:10.1371/journal.pone.0186021)
Supplement: S1 Table — (DOCX) [file pone.0186021.s003.docx]

**Supplementary Table 1.** Primer sequences used in quantitative real-time polymerase chain reaction.

| Gene | Forward Primer Sequnce | Reverse Primer Sequence |
| --- | --- | --- |
| c-fos | ATGGGCTCTCCTGTCAACAC | GACACGGTCTTCACCATTCC |
| c-jun | AAAACCTTGAAAGCGCAAAA | CGCAACCAGTCAAGTTCTCA |
| egr-1 | GAGCGAACAACCCTATGAGC | AGGCCACTGACTAGGCTGAA |
| BNP | CTGAAGGTGCTGTCCCAGAT | CCTTGGTCCTTCAAGAGCTG |
| Acta1 | CCGGGAGAAGATGACTCAAA | CAGGGCATAGCCCTCATAGA |
| Serca2a | CTGTGGAGACCCTTGGTTGT | CAGAGCACAGATGGTGGCTA |
| FOXO3 | CGTTGTTGGTTTGAATGTGG | GAGAGCAGATTTGGCAAAGG |
| PTEN | ACACCGCCAAATTTAACTGC | TACACCAGTCCGTCCCTTTC |
| PPARγ1 | CTGTGAGACCAACAGCCTGA | CAGTGGTTCACCGCTTCTTT |
| CD36 | TGCTGGAGCTGTTATTGGTG | TGGGTTTTGCACATCAAAGA |
| MCAD | AGGTTTCAAGATCGCAATGG | GCGAGCAGAAATGAAACTCC |
| LCAD | GTCACCAACCGTGAAGCTCG | AGGCATTAGCTGGCAATCGG |
| VLCAD | TATCTCTGCCCAGCGACTTT | TGGGTATGGGAACACCTGAT |
| GLUT1 | AAACATGGAACCACCGCTAC | AGGCCAACAGGTTCATCATC |
| GLUT4 | GATTCTGCTGCCCTTCTGTC | ATTGGACGCTCTCTCTCCAA |
| PDK4 | ACCGCATTTCTACTCGGATG | ACTGGTCGCAGAGCATCTTT |

c-Jun-N-terminal kinase, c-jun; early growth response 1, egr-1; brain natriuretic peptide, BNP; actin a1 skeletal muscle, Acta1; sarco/endoplasmic reticulum Ca2+-transport ATPase2a, Serca2a; Forkhead box O3, FOXO3; Phosphatase and tensin homolog, PTEN; peroxisome proliferator-activated receptor γ, PPARγ; cluster of differentiation, CD36; medium-chain acyl-CoA dehydrogenase, MCAD; long-chain acyl-CoA dehydrogenase, LCAD; very long-chain acyl-CoA dehydrogenase, VLCAD; Glucose transporter 1, GLUT1; Glucose transporter 4, GLUT4; pyruvate dehydrogenase kinase 4, PDK4.
